# Right Ovarian Vein Thrombosis in the Setting of COVID-19 Infection

**DOI:** 10.7759/cureus.12796

**Published:** 2021-01-20

**Authors:** Rebecca E DeBoer, Olubunmi O Oladunjoye, Ronald Herb

**Affiliations:** 1 Internal Medicine, Reading Hospital Tower Health, Reading, USA

**Keywords:** covid-19, ovarian vein thrombosis, hypercoagulable state, novel oral anticoagulant

## Abstract

Ovarian vein thrombosis is a rare condition associated with the postpartum state, pelvic disease, gynecological surgeries, and other thrombophilic states. We present the first reported case of right ovarian vein thrombosis (OVT) in the setting of Coronavirus disease 2019 (COVID-19) unrelated to pregnancy, pelvic disease, or surgery. This case highlights the breadth of the hypercoagulable state induced by COVID-19. We also put forward the use of novel oral anticoagulants in the case of OVT.

## Introduction

Ovarian vein thrombosis is an uncommon condition. One report over multiple reviews found that the incidence of ovarian vein thrombosis is approximately 0.05% of vaginal deliveries and 1-2% of cesarean deliveries [1]. A more recent single-center study found the incidence to be even less: just 0.04% or 13 cases out of 40,353 postpartum patients [2]. Furthermore, approximately 90% of the ovarian vein thromboses (OVT) happen in the right ovarian vein [3-4]. It has been reported predominantly in the postpartum state [1-6]. Traditionally, a postpartum or puerperal fever that does not respond to antibiotics should raise suspicion for OVT [6]. Other associated diseases include gynecologic malignancy, pelvic inflammatory disease, and post- gynecologic surgery [3]. However, there is a reported case of OVT associated with Coronavirus disease 2019 (COVID-19) in a pregnant woman [7].

## Case presentation

We present a case of a 56-year-old postmenopausal female with a history of impaired fasting glucose and non-alcoholic fatty liver and no history of thromboembolic disease or recent surgery who came to our office for a transition of care (TOC) appointment. Earlier in the month, she had presented with several days of headache, myalgia, non-productive cough, rhinorrhea, fatigue, chills but had no difficulty breathing. She subsequently tested positive for COVID-19.

**Figure 1:**
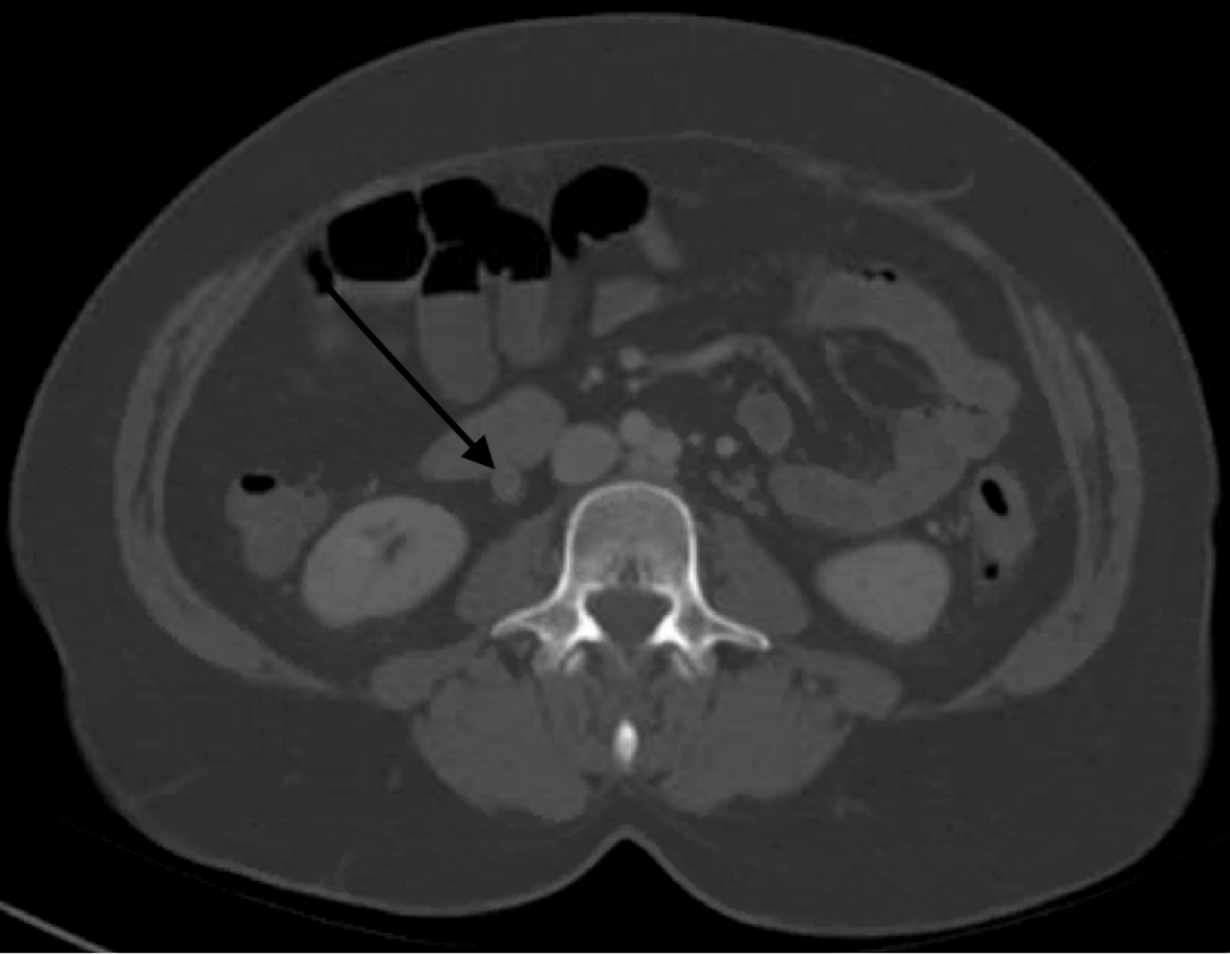
Right ovarian vein with a filling defect (black arrow) consistent with right ovarian vein thrombosis.

Slightly more than one week after the positive test, she developed lower abdominal pain. She presented to the emergency department two days later because of worsening lower abdominal pain. At the time, the physical exam revealed a blood pressure of 141/98mmHg, heart rate 98 beats per minute, temperature 36.2 ºC, respiratory rate 17, and oxygenation 97% on room air. Abdominal examination was positive for tenderness to palpation of the lower quadrant. Otherwise, the rest of the physical examination was unremarkable. Computerized Tomography (CT) scan of the abdomen and pelvis with contrast showed patchy ground-glass opacities throughout the lung bases concerning multifocal pneumonia, and a diffusely enlarged right ovarian vein with a hyperdense central filling defect, consistent with non-occlusive thrombosis. The other structures, including the uterus, were normal with no finding of adnexal masses (Figure 1). Laboratory abnormalities included significantly elevated D-dimer at 18.97 μg/ml (reference range < 0.50 μg/ml), C-reactive protein 5.65 mg/dL (reference range < 1.00 mg/dL), ferritin 632 ng/mL (reference range 11-307 ng/mL), creatine kinase 32 IU/L (reference range 30-223 IU/L), aspartate aminotransferase (AST) 173 IU/L (reference range 13-39 IU/L), alanine transaminase (ALT) 117 IU/L (reference range 7-52 IU/L), white blood cell (WBC) 12.3 x 10^3^/μL (reference range 4.8-10.8 x 10^3^/μL) with lymphocyte number 1.2 x 10^3^/μL (reference range 0.7-5.2 x 10^3^/μL), and lymphocyte percent 8.3 % (reference range 14.0-48.0 %).

Given the finding of right ovarian vein thrombosis, Hematology was consulted. She was started on apixaban 10 mg twice daily for one week followed by apixaban 5 mg twice daily to complete a three-month course of anti-coagulation. The patient subsequently presented to our outpatient office for a TOC visit with the resolution of abdominal pain, normal vital signs, and normal physical examination.

## Discussion

We highlight a case of ovarian vein thrombosis secondary to COVID-19 infection. The patient was postmenopausal and had no prior history of thromboembolic disease. Her only risk factor for thrombosis was the COVID-19 infection. Abdominal pain prompted further evaluation with a CT of the abdomen and pelvis, which revealed ovarian vein thrombosis.

OVT is a rare condition and the exact cause remains unclear. However, it has been attributed to Virchow’s triad: hypercoagulable state, endothelial damage, and stasis [1, 3-4]. Pregnancy as a hypercoagulable state is a well-known risk factor for OVT. Other risk factors in pregnancy theorized to cause OVT include compression of the right ovarian vein and inferior vena cava by the gravid uterus [8] and uterine infection that can cause endothelial damage [1]. The most likely cause of our patient’s right ovarian vein thrombosis was COVID-19 since she had no other risk factors like postpartum state, recent surgeries, or history of thromboembolic disorder.

COVID-19 has been shown to cause hypercoagulability in the form of venous and arterial thromboembolism [9-12]. The pathogenesis of this remains unknown. However, some studies have postulated that patients with COVID-19 may have different coagulation abnormalities due to direct invasion of the endothelial cells by SARS-CoV-2 virus (severe acute respiratory syndrome coronavirus 2) thereby leading to cellular injury. Other sources of endothelial injury also include mediators of acute systemic inflammatory response such as cytokines release [13-14]. In COVID-19, changes in prothrombotic factors like D-dimer, fibrinogen, factor VIII, and von Willebrand factor have been reported to cause a hypercoagulable state. COVID-19 can also present as a critical illness. Critical illnesses have been associated with increased venous stasis due to the use of an intravenous catheter, immobilization, and mechanical ventilation. Since COVID-19 is associated with pro-inflammatory states (elevated C-reactive protein, ferritin, lactate dehydrogenase,) Abou-Ismail et al. proposed that the pathophysiology is centered around the cross-talk between inflammation and thrombosis which is a well-established relationship [9].

Historically, the presentation of OVT is fever and abdominal pain, with tender abdominal mass being less common [4]. In one report on four cases, the women presented with vague abdominal pain, or deep pelvic pain, and fever. Sometimes the pain is localized for example to the right lower quadrant [3, 5, 15].

It has been suggested that imaging modalities to detect OVT can be ultrasound, CT, or magnetic resonance imaging. However, CT seems to be the imaging modality of choice. A radiologic study found that to detect OVT, CT was 100% sensitive and 99% specific [6]. That compares to ultrasound being 50% sensitive and 99% specific and MRI being 92% sensitive and 100% specific [6]. Positive findings on an enhanced CT include filling defect within the wall of the vein, enlargement of the vein, obvious mass, or signs of perivascular edema. One institution’s study used a CT scan to screen for OVT by looking for a filling defect in the afferent ovarian veins [2-3, 6].

Complications that can arise from OVT include an extension of the thrombus itself or pulmonary embolism. Dunnihoo et al. reported pulmonary embolism in 13.2% of patients with postpartum OVT [1, 4]. However, this was not the case in another recent study, perhaps because of early intervention with anticoagulation [2].

Anticoagulation is the mainstay of management, but guideline-directed therapy has not been established. Therapies that have been used include low molecular weight heparin and unfractionated heparin. A study with 13 cases used enoxaparin for a total of three months. Some others have used warfarin. There are no studies to show the efficacy of novel oral anticoagulants in OVT perhaps because the cases are rare for a clinical trial [1-2, 8].

Our patient had presented with vague, lower quadrant abdominal pain without fever and palpable abdominal mass. The diagnosis of OVT was made with a filling defect seen on the CT scan. She was treated as a case of provoked thrombosis and started on apixaban for three months. Her case was not complicated with pulmonary embolism. At the time of follow-up at the TOC visit, abdominal pain had resolved, she denied shortness of breath and examination showed normal vital signs, and adequate oxygenation on room air.

## Conclusions

The diagnosis of right ovarian vein thrombosis should be considered in patients presenting with abdominal pain, especially when they are suffering from hypercoagulable conditions such as the COVID-19 disease, even in the absence of the postpartum state or other risk factors for OVT. Novel oral anticoagulants (NOAC) may also be used for the treatment of OVT. However, further studies on this should be considered in the future.
